# Totally room-temperature solution-processing method for fabricating flexible perovskite solar cells using an Nb_2_O_5_–TiO_2_ electron transport layer†

**DOI:** 10.1039/c8ra01571f

**Published:** 2018-04-03

**Authors:** Jun Jiang, Shubo Wang, Xuguang Jia, Xiang Fang, Shuai Zhang, Jing Zhang, Wei Liu, Jianning Ding, Ningyi Yuan

**Affiliations:** School of Materials Science and Engineering, Jiangsu Collaborative Innovation Center of Photovoltaic Science and Engineering, Jiangsu Province Cultivation Base for State Key Laboratory of Photovoltaic Science and Technology, Changzhou University Changzhou 213164 Jiangsu China dingjn@cczu.edu.cn nyyuan@cczu.edu.cn

## Abstract

Flexible perovskite solar cells are new technology-based products developed by the global solar industry and are promising candidates for realizing a flexible and lightweight energy supply system for wearable and portable electronic devices. A critical issue for flexible perovskite solar cells is to achieve high power conversion efficiency (PCE) while using low-temperature solution-based technology for the fabrication of a compact charge collection layer. Herein, we innovatively introduce niobium ethoxide as a precursor additive to TiO_2_ NCs, which allows realization of an Nb_2_O_5_–TiO_2_ electron transport layer (ETL). The presence of Nb_2_O_5_ remarkably enhances electron mobility and electrical conductivity of the ETLs. In addition, uniform perovskite films are prepared by an annealing-free solution-based method. The excellent performance of the cell is attributed to its smooth film surface and high electron mobility, and performance is verified by the effective suppressions of charge recombination and time-resolved photoluminescence. PCEs of 15.25% and 13.60% were obtained for rigid substrates (glass/fluorine-doped tin oxide) and an indium tin oxide/PET (poly(ethylene terephthalate)) flexible substrate by using a totally room-temperature solution-processing method, respectively.

## Abstract 2

Flexible perovskite solar cells are new technology-based products developed by the global solar industry and are promising candidates for realizing a flexible and lightweight energy supply system for wearable and portable electronic devices.

## Graphical abstract

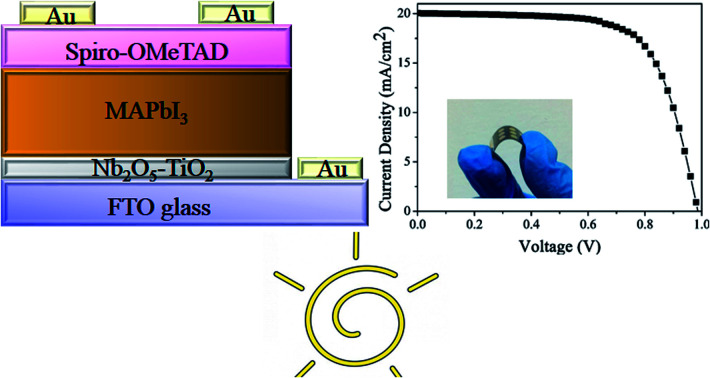

## 1. Introduction

Perovskite solar cells have developed very rapidly over the last several years and could be in considerable demand for various applications in the future photovoltaic market.^1–6^ Numerous scientific research professionals have accelerated increases in power conversion efficiency (PCE) of perovskite solar cells, and at present, the certified world record for a PCE is 22.1%.^7^ As a light absorption layer, APbX_3_ (A = CH_3_NH_3_, (NH_2_)_2_CH_2_ or Cs, and X = I, Br, or Cl) is considered as the most promising replacement for silicon solar cells; APbX_3_ has outstanding properties such as strong light absorption, weakly bound excitons, long-range charge-carrier diffusion, and apparent tolerance to defects.^8–10^

A state-of-the-art photovoltaic solar cell (PSC) is prepared in a conventional n-i-p device configuration consisting of an n-type oxide semiconductor capped with a perovskite absorber and a hole-transport layer (HTL). The electron transfer layer (ETL) of PSCs plays important roles in extracting electrons and blocking holes from perovskite. Metal oxide materials such as TiO_2_, ZnO, and SnO_2_ are widely used in most studies aimed at achieving high efficiency because of their environmentally friendly nature, wide band gap, high electron mobility, and good stability.^11–14^ A large number of PSCs based on TiO_2_ require a high-temperature sintering process for their crystallization or removal of the dispersion medium.^8–10,15^ However, TiO_2_, ZnO, and SnO_2_ ETLs obtained using high-temperature treatment cannot be applied to flexible perovskite solar cells. Recently, methods have been developed for fabricating low-temperature processable TiO_2_ ETLs for planar PSCs, such as atomic layer deposition^16^ and magnetron sputtering.^17^ Compared to vacuum methods, solution-based techniques are generally more cost-effective and scalable and can be used to achieve roll-to-roll process-ability. Therefore, it is imperative to develop effective methods that can restriction migration, improve electron mobility of an ETL, and reduce trap-state density within the perovskite material, thereby eliminating hysteresis and improving the efficiency of PSCs. However, perovskite materials such as CH_3_NH_3_PbI_3_ and CH(NH_2_)_2_PbI_3_ are mostly used for PSCs, and these materials practically need annealing for 10–60 min to form the black crystalline photoactive layer.^18–23^ Annealing process is not convenient for mass production because it requires additional equipment and increases energy consumption. One method to address this problem is to employ annealing-free processing,^15^ which can save energy and facilitate industrial production. Herein, we propose a facile strategy to prepare a highly efficient perovskite solar cell *via* room-temperature solution processing. TiO_2_ films are fabricated by spin coating a colloidal solution of anatase TiO_2_ nanocrystallines (NCs) prepared *via* a low-temperature sol–gel method on a substrate. Considering improved performance of the film, we employed niobium ethoxide as a precursor additive. Nb_2_O_5_ is considered a better ETL for a perovskite solar cell due to its higher carrier mobility and conduction band edge position. Here, niobium ethoxide is introduced into the TiO_2_ dispersion as an aid dispersant. Niobium ethoxide facilitates spontaneous coalescence of the TiO_2_ NCs, thereby forming a stable dispersion. The stable dispersion is spin-coated onto the conductive base film without annealing; only ultraviolet (UV) treatment for 15 min yields a uniform and dense Nb_2_O_5_–TiO_2_ layer. It is expected that the Nb_2_O_5_ formed *in situ* would passivate the grain boundary of the TiO_2_ NCs, and further form a dense and uniform film. The exceptional performance of the layer is attributed to the excellent optical and electronic properties of the Nb_2_O_5_–TiO_2_ material, such as a smooth surface and high electron mobility; these properties make the material a better growth platform for a high-quality perovskite absorber layer. In addition, MAPbI_3_ films are deposited as a light absorption layer *via* one-step spin-coating by a simple annealing-free process.

## 2. Experimental section

### 2.1. Materials

4-*tert*-Butylpyridine (*t*-BP), Li-bis-(trifluoromethanesulfonyl)imide (Li-TFSI), PbI_2_ (99.9985%), niobium ethoxide (99.999%), dimethylacetamide (DMAc) and *N*-methyl-2-pyrrolidone (NMP) were ordered from Alfa Aesar; the methylammonium iodide (MAI) (99.5%), and 2,2′,7,7′-tetrakis[*N*,*N*-di(4-methoxyphenyl)amino]-9,9′-spiro-bifluorene (spiro-OMeTAD) (99.8%) were purchased from Xi'an p-OLED Technology Corp. Titanium tetrachloride (TiCl_4_, anhydrous, 99.5%) and chlorobenzene (anhydrous, 99.9%) were purchased from Aldrich (USA). All solvents were used without any further purification.

### 2.2. Synthesis of TiO_2_ NCs and preparation of TiO_2_ dispersion with niobium ethoxide

TiO_2_ NCs were synthesized by a non-hydrolytic sol–gel reaction according to a modified procedure.^24^ The resulting precipitates were washed by adding excess ethanol and diethyl ether and purified by centrifugation at 3000 rpm for 5 min. This washing procedure was repeated thrice. To obtain the TiO_2_ colloidal solution (∼5 mg mL^−1^), the washed TiO_2_ NCs were dispersed into anhydrous methanol, and ultrasonic treatment was carried out for several hours. To obtain a niobium ethoxide/TiO_2_ mixed precursor suspension for spin coating, the purified TiO_2_ NCs (5 mg mL^−1^) were re-dispersed in ethanol at the desired niobium ethoxide concentration. Nb_2_O_5_–TiO_2_ ETLs were fabricated by spin-coating at 3000 rpm under ambient conditions, and the films were free from annealing. The samples were treated again with UV–ozone for 15 min before perovskite deposition.

### 2.3. Device fabrication

Pre-patterned transparent conducting oxide substrates were sequentially cleaned using ethanol, acetone, isopropanol, and ethanol separately in an ultrasonic bath for 20 min each and then dried under flowing nitrogen. Fluorine-doped tin oxide (FTO) substrates underwent UV–ozone treatment (Model UV-03 UVO_3_ cleaner) for 15 min before they were used for spin-coating ETLs.

A highly dispersed solution of TiO_2_ NCs and TiO_2_ NCs with niobium ethoxide in ethanol were dropped onto substrates and immediately spin-coated at a speed of 3000 rpm for 30 s. The samples again underwent UV–ozone treatment for 15 min before perovskite deposition. Then, the TiO_2_ (Nb_2_O_5_–TiO_2_)-coated substrates were transferred immediately to a nitrogen-filled glovebox for the deposition of perovskite films.

A MAPbI_3_ solution was prepared according to an annealing-free process reported by Fang *et al.*^15^ The MAPbI_3_ precursor solution (1.2 M) was prepared using a mixed solvent of DMAc and NMP in a volume ratio of 5 : 1. Perovskite films were deposited onto the TiO_2_ or Nb_2_O_5_–TiO_2_ substrates according to a two-step spin-coating procedure. In the first step, spin coating was carried out at 1000 rpm for 20 s with an acceleration of 200 rpm s^−1^. In the second step, spin coating was carried out at 5000 rpm for 45 s with an acceleration of 1000 rpm s^−1^. During the second step, chlorobenzene was dropped onto the spinning substrate at 35 s before the end of the procedure. At the end of the second step, a dark perovskite film was directly formed. A spiro-OMeTAD HTL was prepared according to a process reported by Yang *et al.*^25^ The HTL was fabricated as follows: a spiro-OMeTAD solution (90 mg mL^−1^) was dissolved in chlorobenzene using 36 μL 4-*tert*-butylpyridine and 22 μL lithium bis(trifluoromethylsulfonyl)imide (520 mg mL^−1^) as the dopants in acetonitrile. The spiro-OMeTAD solution was spin-coated onto the perovskite films at 3000 rpm for 30 s. Finally, an 80 nm thick gold coating was deposited using a thermal evaporator.

### 2.4. Characterization

Morphologies of the TiO_2_ films were characterized by field-emission scanning electron microscopy (FESEM, Zeiss Supra 55). The *ζ*-potential of the TiO_2_ NCs with different concentrations of niobium ethoxide was characterized by using a size analyzer (Zetasizer Nano ZS ZEN3600 instrument, Malvern Instruments) at room temperature with a 633 nm laser. The UV-vis transilluminator spectra of the samples were recorded on a spectrophotometer (UV5800). High-resolution transmission electron microscopy (HR-TEM) was carried out using an electron microscope (JEM-2100, JEOL Ltd., Japan). The root-mean-square (RMS) roughness and topography images of the films were obtained *via* atomic force microscopy (AFM, Veeco Dimension V). The quality and crystalline structure of the samples were confirmed by *θ*–2*θ* X-ray diffraction (XRD) using an X-ray diffractometer (D/max 2500 PC) with a Cu Kα radiation source. A photoluminescence (PL) system (DeltaFlex, Horiba Ltd.) was used to measure the time-resolved PL (TRPL) decay. Photovoltaic performance of the solar cells was measured using a multisource meter (Model 2400, Keithley, Cleveland, OH, USA) under one sun (AM 1.5G, 100 mW cm^−2^) illumination, which was achieved by using a solar simulator (500 W Xe lamp) (XES-40S1, San-Ei Electric Co., Ltd., Japan) as the light source. The device area of 0.07 cm^2^ was defined by a metal mask. All devices were scanned with a reverse and forward under standard test procedure at a scan rate of 0.2 V s^−1^. X-Ray photoelectron spectroscopy (XPS) was performed on a photoelectron spectrometer (ESCALAB 250Xi, Thermo Fisher Scientific).

## 3. Results and discussion

### 3.1. Characterization of TiO_2_ and Nb_2_O_5_–TiO_2_ films

**Fig. 1:**
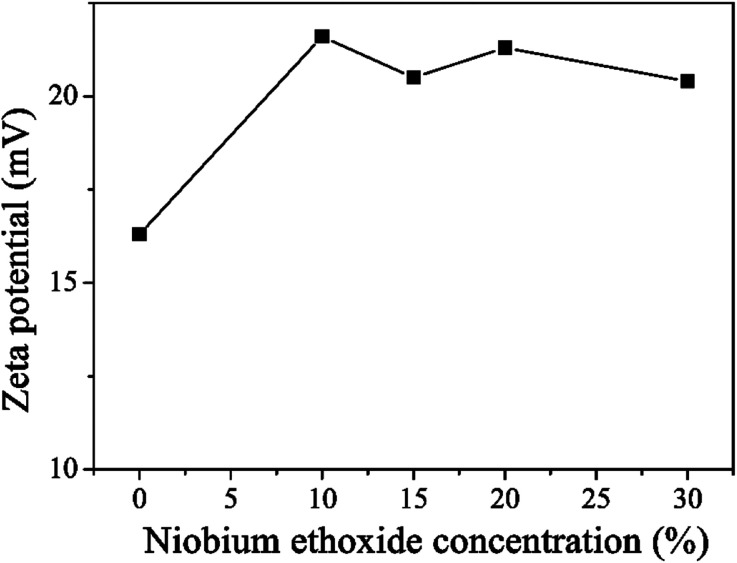
The zeta potential of TiO_2_ NCs with different concentration of niobium ethoxide.

**Fig. 2:**
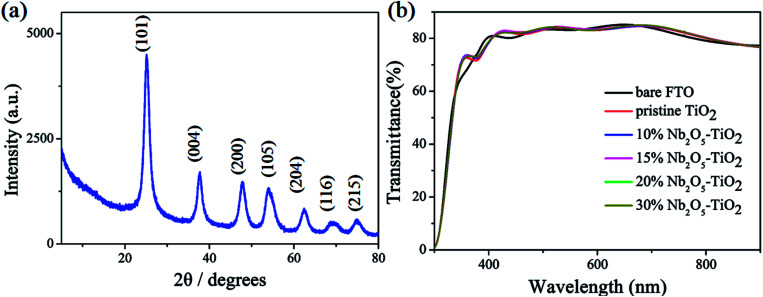
(a) Powder X-ray diffraction pattern of TiO_2_ NCs. (b) Transmittance spectra of bare FTO, pristine TiO_2_, and Nb_2_O_5_–TiO_2_ with different niobium ethoxide contents.

In the spin-coating process, a stably distributed precursor solution of TiO_2_ NCs in solution is a prerequisite to form uniform and void-free TiO_2_ thin films. The niobium ethoxide-capped TiO_2_ NCs have a uniform dispersion in ethanol, are stable for months, and have better anti-settleability properties (Fig. S1†). The *ζ*-potential is an important factor reflecting colloid stability. The *ζ*-potential was measured, and the results are shown in Fig. 1. The figure indicates that the *ζ*-potential value for TiO_2_ is 16.3 mV. The *ζ*-potential increases with the addition of niobium ethoxide. It demonstrated that the niobium ethoxide plays an important role in the dispersion of TiO_2_ NCs. Powder XRD measurements of a TiO_2_ sample revealed typical diffraction peaks of anatase TiO_2_ (Fig. 2a). The diffraction peaks centered at 25.063, 37.682, 47.882, 54.119, 62.418, and 75.029 are assigned to (101), (004), (200), (105), (204), (116), and (215) diffractions, respectively. This result confirms that the as-synthesized TiO_2_ NCs are assigned to the anatase crystal structure (PDF no. 21-1272). Scherrer peak width analysis^26^ revealed that the average size of the nanocrystalline domains is approximately 8.8 nm. To gain further insights into the nanoscale morphology and nanocrystal structures, the morphology of as-synthesized TiO_2_ NCs was investigated using the HR-TEM images. The HR-TEM images (Fig. S2a†) revealed that the TiO_2_ NCs are around 5–10 nm in diameter, which is consistent with the value obtained by Scherrer peak width analysis. In addition, the selected area electron diffraction pattern for the TiO_2_ NCs (Fig. S2b†) confirms the high crystallinity of the TiO_2_ NCs.

Fig. 2b shows the optical transmission spectra of TiO_2_ and Nb_2_O_5_–TiO_2_. Both the materials show excellent transmittance in the wavelength range of 400–800 nm. The resultant ETLs coated on the FTO glass substrates shows good optical transparency and higher transmittance than the bare FTO glass, with the transmittance being greater than 80% in the entire visible region. The high transparency of the Nb_2_O_5_–TiO_2_ ETLs is very conducive to the light absorption of the perovskite layer and improves light harvesting. No apparent difference was found between the transmittances of different Nb_2_O_5_–TiO_2_ films deposited on the FTO substrates.

**Fig. 3:**
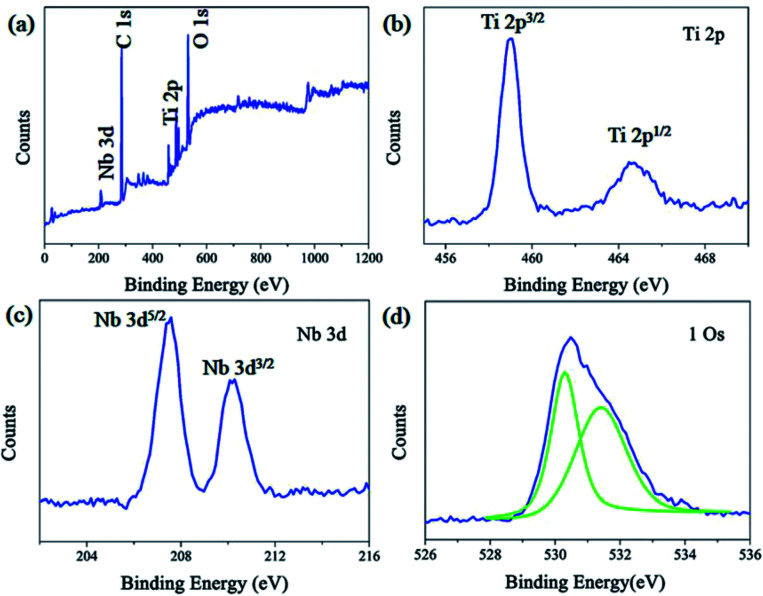
(a) Typical XPS survey of Nb_2_O_5_–TiO_2_ film. High-resolution XPS (b) Ti 2p, (c) Nb 3d, and (d) O 1s peaks of the Nb_2_O_5_–TiO_2_ film deposited on a glass substrate.

The composition and bonding type of the Nb_2_O_5_–TiO_2_ film were measured using XPS. A typical XPS spectra of Nb_2_O_5_–TiO_2_ is shown in Fig. 3a. Clearly, the O, Ti, and Nb peaks are located at ∼530.59, ∼458.99, and ∼207.51 eV, respectively. The high-resolution Ti 2p (Fig. 3b) spectrum reveals two different peaks located at 459.04 and 464.69 eV, which correspond to Ti 2p^3/2^ and Ti 2p^1/2^, respectively; accordingly, a spin–orbit coupling of 5.65 eV is obtained, which is the signature of Ti^4+^. As shown in the Nb 3d core level spectra (Fig. 3c), Nb 3d^5/2^ and Nb 3d^3/2^ peaks are located at 207.54 and 210.20 eV, respectively, indicating the presence of five-valent niobium in the deposited films.^27^ The main binding energy of 530.3 eV is attributed to O 1s (Fig. 3d) which indicates the O^2−^ state in TiO_2_ and the peak at the higher binding energy of 531.4 eV is attributed to surface oxygen-group absorbance or hydroxyl groups.^28–31^

**Fig. 4:**
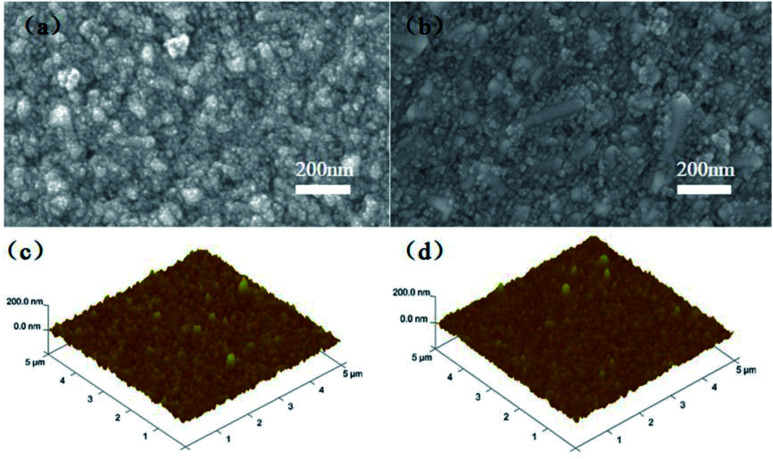
Top-view SEM images of (a) TiO_2_ and (b) Nb_2_O_5_–TiO_2_ films coated on FTO substrates. AFM height images of the (c) TiO_2_ and (d) Nb_2_O_5_–TiO_2_ films.

Fig. 4a and b show the top-view SEM images of the TiO_2_ and Nb_2_O_5_–TiO_2_ films. The Nb_2_O_5_–TiO_2_ film is uniform and dense and shows no apparent pinholes, indicating the high quality of the film. The surface morphology of the Nb_2_O_5_–TiO_2_ film is not like to that of the pristine TiO_2_ film. The results of simultaneous energy-dispersive spectroscopy (EDS) of the Nb_2_O_5_–TiO_2_ film are shown in Fig. S3.† When excess niobium ethoxide (30% and 40%) is added, the surface morphology indicates the formation of a porous surface with a large number of pinholes, directly causing a deterioration in the quality of the thin film (Fig. S4†). Fig. 4c and d shows the AFM height images of the TiO_2_ and Nb_2_O_5_–TiO_2_ films. The RMS roughness decreased from 13.8 to 10.5 nm because of the introduction of Nb_2_O_5_, indicating that the Nb_2_O_5_–TiO_2_ films have flat surfaces. A smooth surface is essential for growing high-quality perovskite films, reducing surface defect trap interfaces with ETLs and HTLs, and enhancing charge extraction at the interface between the ETL and the perovskite layer.^32–34^

To investigate the effect of introducing Nb_2_O_5_ on the electrical properties, the electron mobility was studied by the space charge limited current (SCLC) method using an electron-only device structure. The sample structure used for this measurement was FTO/PCBM/TiO_2_ (15% Nb_2_O_5_–TiO_2_)/PCBM/Ag. The details are shown in the ESI.† Fig. S5† shows the current density–voltage (*J*–*V*) curves for the TiO_2_ and Nb_2_O_5_–TiO_2_ films fitted using the Mott–Gurney law.^34,35^ It's apparent that the electron mobility of the ETL film increases considerably because of the introduction of Nb_2_O_5_. The electron mobility increases from 7.09 × 10^−4^ to 1.14 × 10^−3^ cm^2^ V^−1^ s^−1^, which is very close to previous reports that TiO_2_ modified craft with annealing process.^36^

### 3.2. Photovoltaic performance

**Fig. 5:**
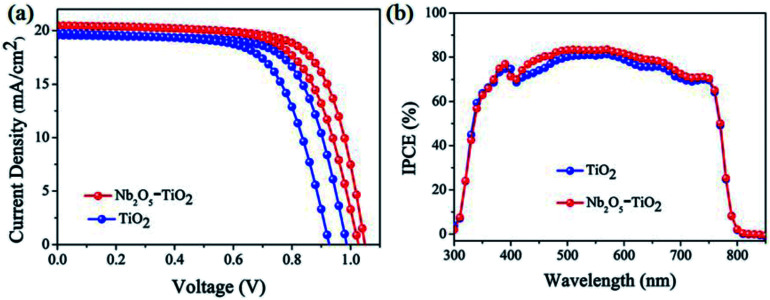
(a) *J*–*V* curves and (b) IPCE spectra of the best-performing PSCs made with TiO_2_ and Nb_2_O_5_–TiO_2_ ETLs on FTO glass.

**Fig. 6:**
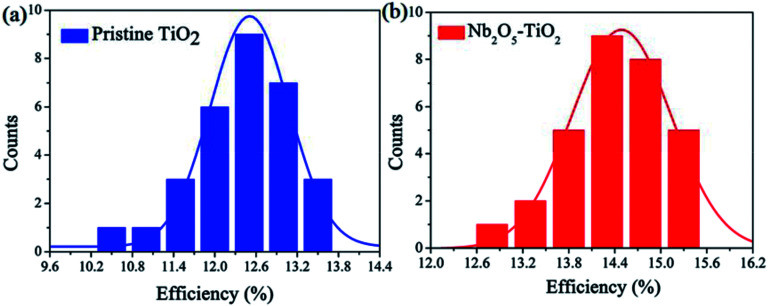
PCE distribution histogram of PSCs based on TiO_2_ (a) and Nb_2_O_5_–TiO_2_ (b) ETLs.

The above measurements show that the quality of the TiO_2_ NCs film improves considerably because of the introduction of Nb_2_O_5_; *e.g.*, the film has a smoother surface and enhanced electron mobility. Next, PSCs were designed and fabricated using the TiO_2_ and Nb_2_O_5_–TiO_2_ ETLs. Fig. S6† shows the detailed device structure, in which FTO is employed as the anode, the TiO_2_ or Nb_2_O_5_–TiO_2_ film as the ETL, MAPbI_3_ as the absorber layer, spiro-OMeTAD as the HTL, and a gold layer as the cathode. A cross-sectional SEM image of the completed device architecture is shown in Fig. S7.† The smooth morphology of the Nb_2_O_5_–TiO_2_ films is beneficial for forming highly crystalline and compact perovskite films. Fig. S8† shows the top-view SEM images of the perovskite film, which exhibits smooth surfaces, big crystalline size, and good coverage. Table S1† lists the device performance optimized as a function of the Nb_2_O_5_ content; the key *J*–*V* parameters are summarized in ESI.† It appears that the PCE increased from 13.47% to 15.25%. It is apparent that at the optimum Nb_2_O_5_ content, the device performance reaches over 15%. The corresponding photovoltaic parameters for the champion cells are summarized in Table 1. The PSCs based on TiO_2_ ETLs have a short-circuit current density (*J*_sc_) of 19.55 mA cm^−2^, an open circuit voltage (*V*_oc_) of 0.99 V, a fill factor (FF) of 0.698, and a PCE of 13.47%. Compared to the TiO_2_ ETL-based PSC, the Nb_2_O_5_–TiO_2_ ETL-based PSC has enhanced parameter values: *J*_sc_ is 20.49 mA cm^−2^, *V*_oc_ is 1.04 V, FF is 0.716, and the PCE is 15.25%, which is the best efficiency achieved in this study. Compared to the TiO_2_ ETL-based device, all the key *J*–*V* parameters of the Nb_2_O_5_–TiO_2_ ETL-based device are considerably better. Fig. 5a shows the *J*–*V* curves for the champion devices based on both TiO_2_ and Nb_2_O_5_–TiO_2_ ETLs measured in the reverse and forward scan directions. Compared to the control device, the photovoltaic performance of these champion devices is considerably better; the larger *J*_sc_ and FF are attributed to the improved electron mobility and better hole blocking effect of the Nb_2_O_5_–TiO_2_ ETL, and the high *V*_oc_ may be due to the reduced charge recombination and improved electron extraction.^37–39^Fig. 5b shows the incident photon-to-current efficiency spectra for various ETLs. The integrated current density value for the pristine TiO_2_-based cell is 18.92 mA cm^−2^, and it increases to 19.50 mA cm^−2^ for the Nb_2_O_5_–TiO_2_-based device; that value is in good agreement with the *J*–*V* measurement value. Performance statistics for 30 individual cells with TiO_2_ and Nb_2_O_5_–TiO_2_ ETLs are shown in Fig. 6. Clearly, PCEs show a narrower distribution with a smaller standard deviation for the Nb_2_O_5_–TiO_2_-based cells, indicating good reproducibility.

**Table 1:** Key parameters of champion PSCs based on pristine TiO_2_ and Nb_2_O_5_–TiO_2_ ETLs

	Scan direction	*V*_oc_ (V)	*J*_sc_ (mA cm^−2^)	FF	PCE (%)
Nb_2_O_5_–TiO_2_	Reverse	1.04	20.49	0.716	15.25%
	Forward	1.02	20.47	0.678	14.17%
TiO_2_	Reverse	0.99	19.55	0.698	13.47%
	Forward	0.93	19.76	0.664	12.17%

### 3.3. Recombination

**Fig. 7:**
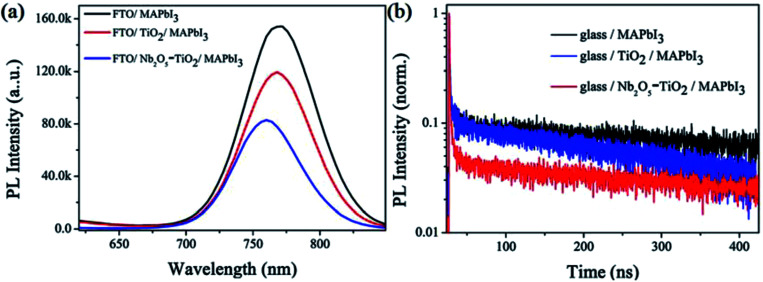
(a) Steady-state photoluminescence (PL) spectra and (b) normalized time-resolved photoluminescence (TRPL) spectra of pristine perovskite and perovskite layers grown on TiO_2_ and Nb_2_O_5_–TiO_2_ ETLs.

To gain insight into the electron extraction and transport mechanism, the steady-state PL and TRPL were measured for the perovskite absorber layer deposited on both the ETL-based substrates. Fig. 7a shows that the spectral peak for the FTO/perovskite sample at 766 nm apparently has the highest PL intensity, indicating serious recombination occurring in the sample. The FTO/Nb_2_O_5_–TiO_2_/perovskite sample has the lowest PL intensity, even lower than that of the FTO/TiO_2_/perovskite sample. Interestingly, compared to the PL peak for the control samples, the peak for the FTO/Nb_2_O_5_–TiO_2_/perovskite film exhibits a considerable blue-shift.^40^ This suggests that Nb_2_O_5_–TiO_2_ substrate is favorable to obtain higher-quality perovskite films than the control group. Perovskite with higher crystallinity and considerably less trap density than the corresponding bandgap will decrease (*i.e.*, the conduction band minimum will move down).^41^Fig. 7b shows the time-resolved photoluminescence (TRPL) decay curves obtained to analyze the PL lifetimes of the perovskite films prepared on both the ETL-based substrates. PL decay transients and the corresponding PL lifetimes were obtained by performing fitting using a bi-exponential decay function, *f*(*t*) = *A*_1_ exp(−*t*/*τ*_1_) + *A*_2_ exp(−*t*/*τ*_2_), where *τ*_1_ is the fast transient component representing the surface properties and *τ*_2_ is the slow component resulting from the bulk properties.^42,43^ The fitted parameters for the glass/perovskite, glass/TiO_2_/perovskite, and glass/Nb_2_O_5_–TiO_2_/perovskite samples are summarized in Table S2.† For the glass/CH_3_NH_3_PbI_3_ sample, *τ*_1_ is 1.05 ns (1.89%) and *τ*_2_ is 429.50 ns (98.11%), with an amplitude average lifetime of 49.40 ns. For the TiO_2_/CH_3_NH_3_PbI_3_ sample, *τ*_1_ is 1.36 ns (2.93%) and *τ*_2_ is 255.33 ns (97.07%), with an amplitude average lifetime of 39.48 ns. For the Nb_2_O_5_–TiO_2_/CH_3_NH_3_PbI_3_ sample, *τ*_1_ is 0.92 ns (11.68%) and *τ*_2_ is 223.58 ns (88.32%), with an average lifetime was 7.60 ns. Therefore, it is apparent that the photon-induced electrons transfer from perovskite to the Nb_2_O_5_–TiO_2_ ETL is faster and more effective than that from perovskite to the TiO_2_ ETL. Thus, the PL characterizations confirm that the large enhancement in the photovoltaic performances of the Nb_2_O_5_–TiO_2_ ETL can be attributed to the enhanced charge extraction.

### 3.4. Flexible perovskite solar cells

**Fig. 8:**
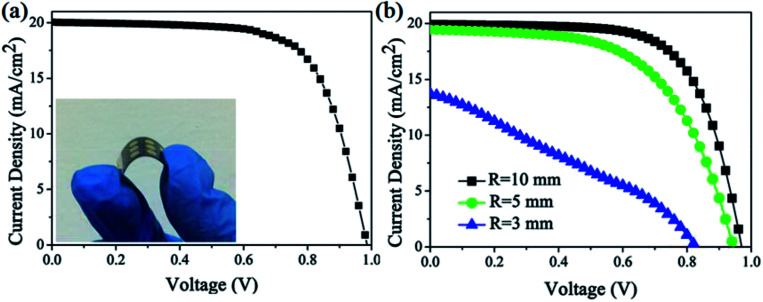
(a) The *J*–*V* curves of best PCE of the perovskite solar cells device using flexible PET/ITO substrates. The inset shows the flexible PSC based on Nb_2_O_5_–TiO_2_ ETLs coated on ITO/PET substrate. (b) *J*–*V* curves of the flexible PSCs obtained after recovery from bending at various radius (*R* = 10, 5, and 3 mm).

The totally room-temperature UV process for efficient ETLs is very suitable for fabricating high-performance flexible PSCs. The excellent performance of uniform and dense Nb_2_O_5_–TiO_2_ ETLs prompted us to fabricate flexible perovskite solar cells using flexible substrates. Nb_2_O_5_–TiO_2_ ETLs were successfully fabricated on indium tin oxide (ITO)/PET substrates, and perovskite, spiro-OMeTAD, and a gold electrode were sequentially deposited using the same methods as those used for the FTO glass-based devices. Fig. 8a shows the *J*–*V* curves for the perovskite solar cells fabricated using flexible ITO/PET substrates, and the inset illustrates a photograph of a flexible perovskite solar cell fabricated using flexible ITO/PET substrates. For the flexible device, *J*_sc_ is 20.04 mA cm^−2^, *V*_oc_ is 0.99 V, and the FF is 0.69, giving the best PCE of 13.60%. The PCE is lower than that of the rigid device because of the decreased *J*_sc_, *V*_oc_ and FF; the decrease is probably caused by the higher series resistance and lower transmittance of the ITO/PET substrate in the short wavelength spectrum.^17^Fig. 8b shows the *J*–*V* curves for the flexible device after it is recovered from the given bending radius. The key *J*–*V* parameters of the devices are summarized in Table S3.† After the device is bent with *R* = 10, 5, and 3 mm, the PCE values degenerate to 13.03%, 10.70%, and 3.39%, respectively. As shown in Fig. 8b, the performance of the flexible device does not show serious degradation when the bending radius is 10 mm. When the bending radius is 3 mm, the brittle ITO breaks^44^ and the PCE is greatly reduced simultaneously, indicating that the flexible devices show good mechanical stability and the Nb_2_O_5_–TiO_2_ ETLs is a promising electron transport material.

## 4. Conclusion

We have demonstrated that Nb_2_O_5_–TiO_2_ is an excellent ETL material for perovskite solar cells, with the champion cell showing a considerably higher PCE (15.25%) than that of devices based on a pristine TiO_2_ ETL and that of a rigid substrate (13.47%). In the proposed process, niobium ethoxide facilitates the spontaneous coalescence of the TiO_2_ NCs, thereby forming Nb_2_O_5_–TiO_2_ ETL. Our results suggest that low-temperature solution-processed Nb_2_O_5_–TiO_2_ could be a good ETL candidate for producing efficient perovskite solar cells. Our facile strategy is highly suitable for fabricating high-performance flexible PSCs because it does not need a high-temperature process and can easily modify the ETL *via* the direct addition of a reagent. This approach will pave the way for further advances in flexible PSCs and is feasible for large scale roll-to-roll processing.

## Conflicts of interest

There are no conflicts to declare.

## Supplementary Material

RA-008-C8RA01571F-s001
