# Supplementary material for: Totally room-temperature solution-processing method for fabricating flexible perovskite solar cells using an Nb2O5–TiO2 electron transport layer
Source: RSC Adv. 2018 Apr 3;8(23):12823–31. doi: 10.1039/c8ra01571f (PMC9079622; doi:10.1039/c8ra01571f)
Supplement: RA-008-C8RA01571F-s001 [file RA-008-C8RA01571F-s001.pdf]

Electronic Supplementary Material (ESI) for RSC Advances.
This journal is © The Royal Society of Chemistry 2018

Supplementary Information

Totally Room-temperature Solution-Processing Method for Fabricating Flexible Perovskite Solar Cells Using Nb_2O_5-TiO_2 Electron Transport Layer

Jun Jiang, Shubo Wang, Xuguang Jia, Xiang Fang, Shuai Zhang, Jing Zhang, Wei Liu, Jianning Ding*, Ningyi Yuan*

School of Materials Science and Engineering, Jiangsu Collaborative Innovation Center of Photovoltaic Science and Engineering, Jiangsu Province Cultivation Base for State Key Laboratory of Photovoltaic Science and Technology, Changzhou University, Changzhou 213164, Jiangsu China.
* Corresponding authors: dingjn@cczu.edu.cn; nyyuan@cczu.edu.cn

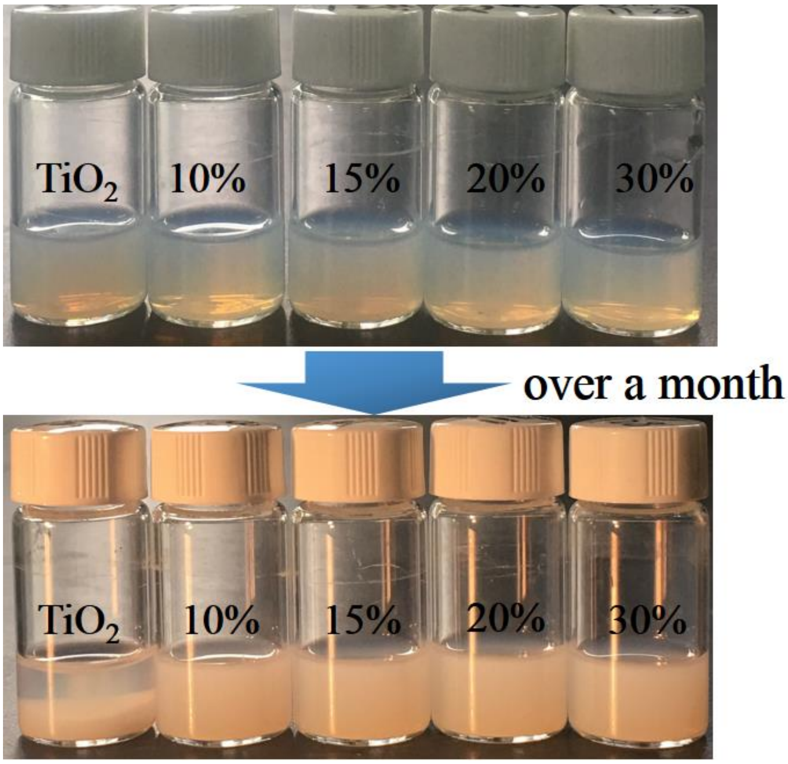

Fig. S1 Photo of TiO_2 particle distribution results from adding different concentrations of niobium ethoxide.

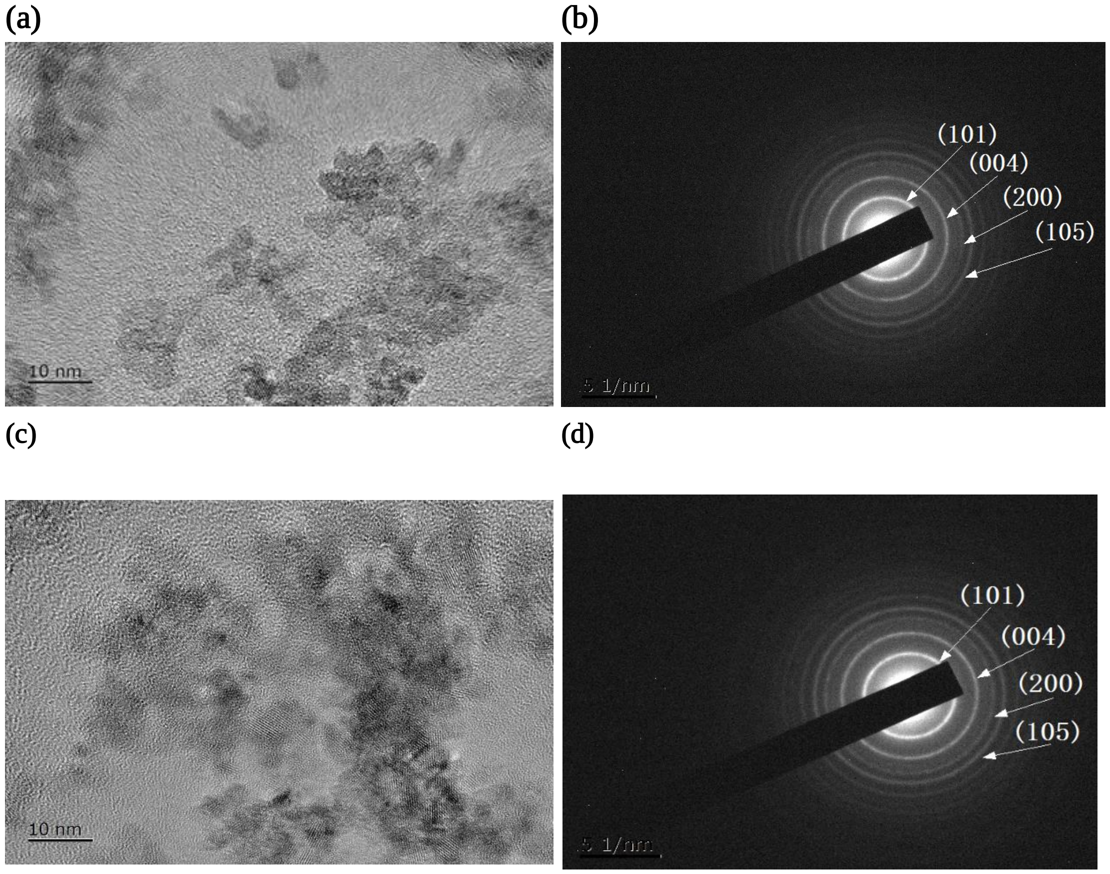

Fig. S2 (a) Bright-field HRTEM image of TiO_2 nanocrystals deposited on a carbon film. (b) A selected area electron diffraction of TiO_2 NCs. (c) Bright-field HRTEM image of Nb_2O_5-TiO_2 nanocrystals deposited on a carbon film. (d) A selected area electron diffraction of Nb_2O_5-TiO_2 NCs.

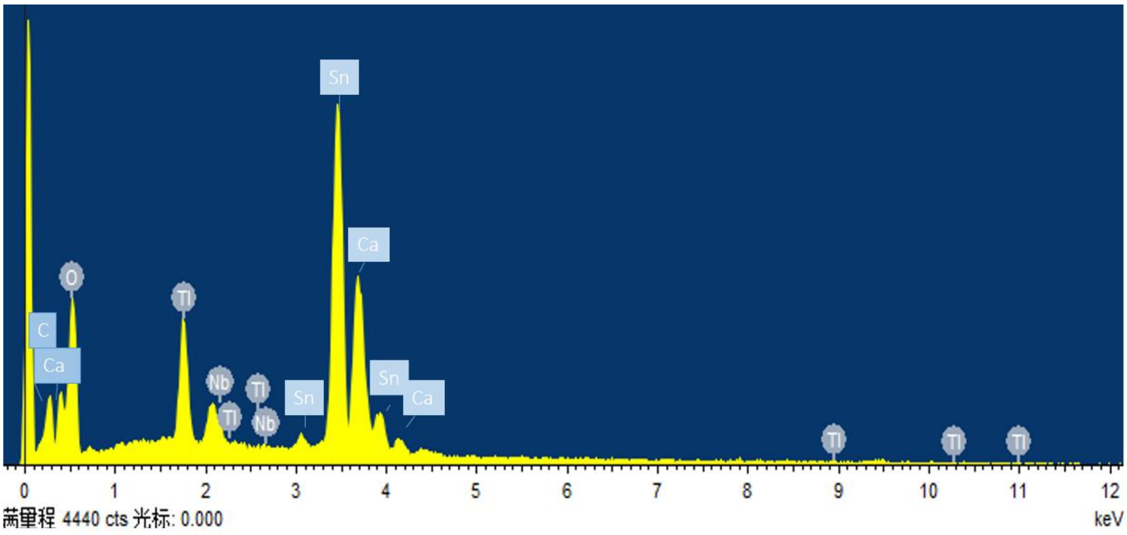

Fig. S3 Energy-dispersive spectroscopy (EDS) of the Nb_2O_5-TiO_2 film.

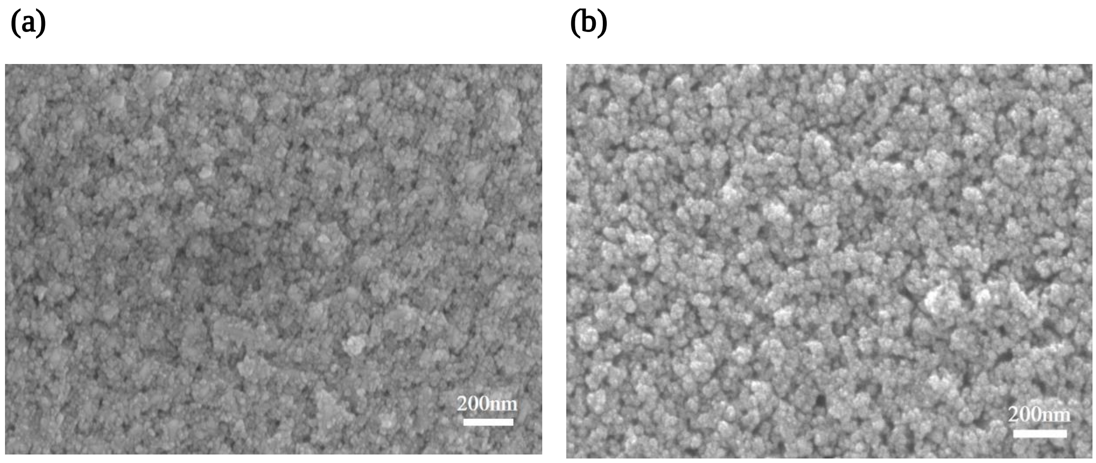

Fig. S4 SEM image of TiO_2 NCs films with over concentrations of niobium ethoxide (a) concentration of 30% and (b) concentration of 40%.

Electron mobility of TiO_2 and Nb_2O_5-TiO_2

Electron-only devices (Fig. S5b) were fabricated to calculate the electron mobility of the samples, including TiO_2 and Nb_2O_5-TiO_2 by the SCLC. The PCBM solution was prepared in chlorobenzene (20 mg/mL), and spin-coated on FTO/glass at 3000 rpm, then annealed at 100 ℃ for 15 min. The TiO_2 and Nb_2O_5-TiO_2 were deposited on PCBM surface. Sequentially, the PCBM films were fabricated on previous samples surface. The 100 nm-thick Ag were deposited. The dark *J-V* characteristics of the electron-only devices were measured by a Keithley 2400 source. The mobility is extracted by fitting the *J-V* curves by the Mott-Gurney.[1, 2]

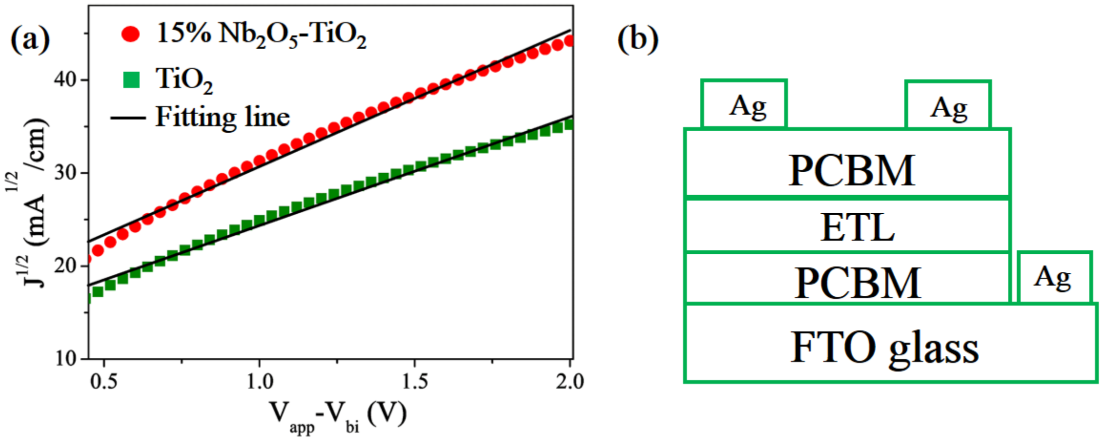

Fig. S5 (a) The electron mobility measurement of TiO_2 and Nb_2O_5-TiO_2 from the space charge limitation of current (SCLC) *J-V* characteristics obtained in the dark for electron-only devices. (b) The sample structure for this measurement.

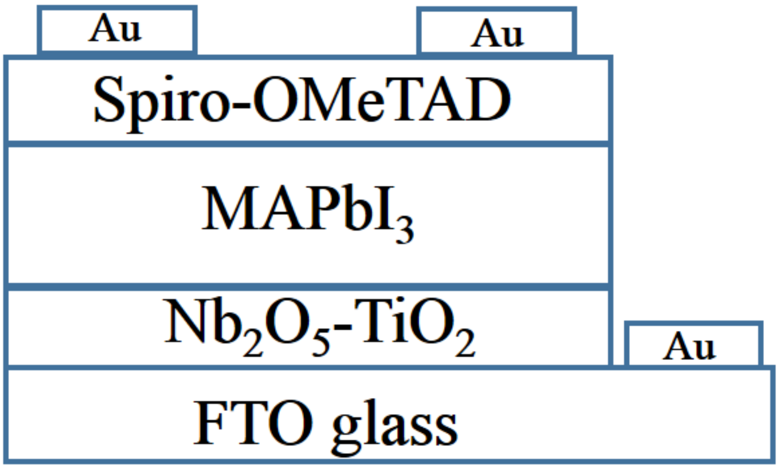

Figure S6 Device structure of the PSC.

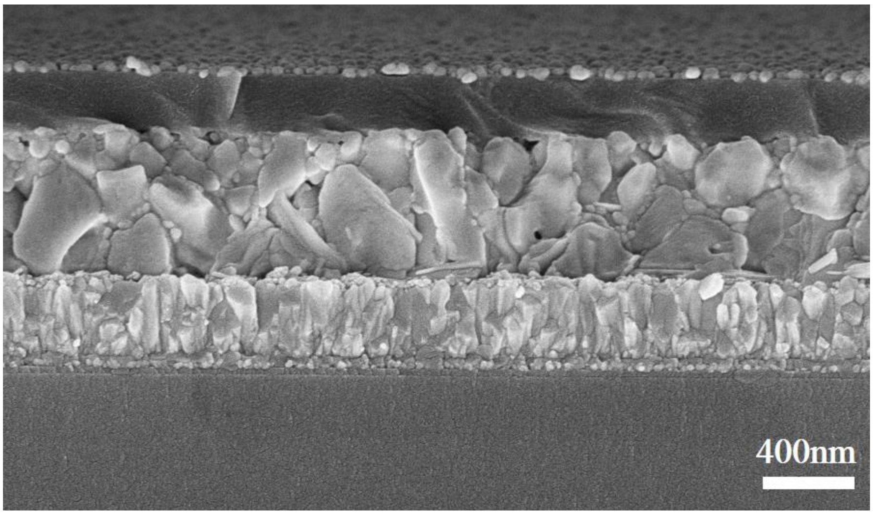

Fig. S7 Cross-sectional SEM image of the PSC completed device.

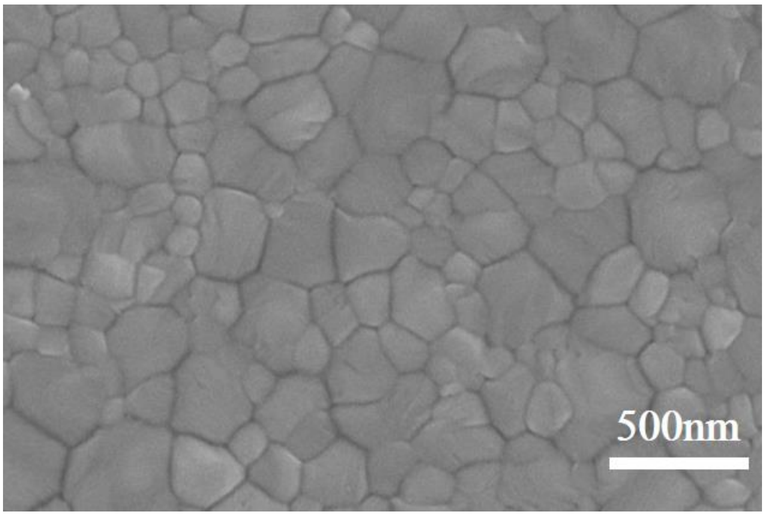

Fig. S8 Top-view SEM images of $CH_3NH_3PbI_3$ perovskite films coated on the Nb_2O_5-TiO_2 films.

	V_{oc}(V)	*J*sc (mA/cm^2)	FF (%)	PCE (%)	Rs (Ω・cm^2)	Rsh (KΩ・cm^2)
TiO_2	0.99	19.55	0.698	13.47	8.12	1.30
10% Nb_2O_5-TiO_2	1.00	20.14	0.711	14.27	7.46	2.61
15% Nb_2O_5-TiO_2	1.04	20.49	0.716	15.25	7.11	2.61
20% Nb_2O_5-TiO_2	1.04	20.13	0.701	14.56	7.37	1.32
30% Nb_2O_5-TiO_2	1.01	20.26	0.648	13.28	8.51	1.19
40% Nb_2O_5-TiO_2	1.00	19.56	0.604	11.86	10.71	0.62

Table S1 Photovoltaic performance of TiO_2 and Nb_2O_5-TiO_2 ETLs

	Amplitude of τ1 (%)	τ1(ns)	Amplitude of τ2 (%)	τ2(ns)	τ_{ave}(ns)
glass/perovskite	1.89	1.05	98.11%	429.50	49.40
TiO_2/ perovskite	2.93%	1.36	97.07%	255.33	39.48
Nb_2O_5-TiO_2 / perovskite	11.68%	0.92	88.32%	223.58	7.60

Table S2 Time resolved photoluminescence (TRPL) parameters based on the glass/perovskite, TiO_2/ perovskite and Nb_2O_5-TiO_2 / perovskite, respectively.

	V_{oc}(V)	*J*sc (mA/cm^2)	*FF* (%)	*PCE* (%)
R=∞	0.99	20.04	0.69	13.60
R=10mm	0.97	19.92	0.67	13.03
R=5mm	0.94	19.45	0.58	10.70
R=3mm	0.82	13.75	0.30	3.39

Table S3 Photovoltaic parameters of the flexible PSCs obtained after recovery from bending at different radius (R).

References

(1) Goodman, A. M.; Rose, A. Double Extraction of Uniformly Generated Electron-Hole Pairs from Insulators with Noninjecting Contacts, J. Appl. Phys. **1971**, 42(7), 2823-2830.

(2) Goh, C.; Kline, R. J.; McGehee, M. D.; Kadnikova, E. N.; Frechet, J. M. J. Molecular-weight-dependent mobilities in regioregular poly(3-hexyl-thiophene) diodes, Appl Phys. Lett. **2005**, 86 (12), 122110.
